# Heavy Metals Affect the Antioxidant Defences in the Soil Ciliate *Rigidohymena tetracirrata*

**DOI:** 10.3390/jox15050169

**Published:** 2025-10-17

**Authors:** Govindhasamay R. Varatharajan, Antonio Calisi, Santosh Kumar, Daizy Bharti, Arnab Ghosh, Shikha Singh, Amit C. Kharkwal, Martina Coletta, Francesco Dondero, Antonietta La Terza

**Affiliations:** 1 School of Biosciences and Veterinary Medicine, University of Camerino, Via Gentile III da Varano, 62032 Camerino, MC, Italy; varatharajangr@smbu.edu.cn (G.R.V.); daizybharti83@gmail.com (D.B.); martina.coletta@unicam.it (M.C.); 2 Faculty of Biology, Shenzhen MSU-BIT University, International University Park Road, Dayun New Town, Longgang District, Shenzhen 518172, China; 3 Department of Sciences and Innovative Technology, University of Eastern Piedmont, Viale Teresa Michel 11, 15121 Alessandria, AL, Italy; antonio.calisi@uniupo.it (A.C.); francesco.dondero@uniupo.it (F.D.); 4 Zoological Survey of India, Prani Vigyan Bhawan, M-Block, New Alipore, Kolkata 700053, India; santoshkumar@zsi.gov.in (S.K.); write2aghosh@gmail.com (A.G.); 5 Amity Institute of Microbial Technology, Amity University Uttar Pradesh (AUUP), Noida 201313, India; shikhasiingh23@gmail.com (S.S.); ackharkwal@amity.edu (A.C.K.)

**Keywords:** acute toxicity, heavy metals, bimetallic mixtures, antioxidant, TPC, DPPH

## Abstract

In this study, we evaluated the cytotoxicity and antioxidant activity of the soil ciliate *Rigidohymena tetracirrata* (Gellért, 1942) Berger 2011, exposed to single and bimetallic mixtures of heavy metals (HMs) for 24 h. Ecotoxicological tests showed LC_20_ values of 0.16, 19.86 and 0.68 mg L^−1^ to Copper (Cu), Zinc (Zn), and Cadmium (Cd), respectively, and LC_50_ values of 0.25, 44.12 and 1.12 mg L^−1^, respectively. Furthermore, it was observed that the mixture of Cd and Zn exhibited antagonism in comparison to other mixtures, (Cd + Cu and Cu + Zn). In the total phenolic content (TPC) assay, a higher phenolic content was observed for the LC_20_ of extracellular Cu (*p* ≤ 0.01) and the LC_20_ of intracellular Cd (*p* ≤ 0.001). The LC_50_ values for Cd and Zn in both extracellular and intracellular contents demonstrated increased α,α-diphenyl-β-picrylhydrazyl (DPPH) scavenging activity with significant values of *p* ≤ 0.05, respectively. Regarding hydroxyl scavenging activity (HRSA), the LC_50_ of extracellular Cd (*p* ≤ 0.001) and LC_50_ of intracellular Cu (*p* ≤ 0.001) exhibited higher antioxidant activity. Therefore, the present study suggests that *R. tetracirrata* holds considerable potential as bioindicators and could be used as a model organism in ecotoxicological studies of soil polluted by HMs.

## 1. Introduction

Soil pollution is one of the most significant problems facing the ecosystems and has enormously increased during the last decades due to intensive human activities (use of biocides and fertilisers, industries, urban waste and atmospheric deposition) [1,2]. Soil pollution causes harm to soil fertility, soil structure, flora and fauna residing in the soil, crops, and groundwater contamination [2]. In soil ecosystems, different types of pollutants exist, with one of the most predominant issues being the contamination of soil by heavy metals [2,3]. Metals are continuously released into the soil by emissions from the rapidly expanding industrial areas, mine tailings, disposal of high metal wastes, leaded gasoline and paints, the land application of fertilisers (agriculture), animal manures, sewage sludge, pesticides, wastewater irrigation, the spillage of petrochemicals, and atmospheric deposition [1,2,3,4,5].

The heavy metal contamination may change the soil properties, especially soil biological properties [6]. The toxicity of heavy metals on soil organisms depends on several factors such as soil temperature, pH, clay minerals, organic matter, inorganic anions and cations, and chemical forms of the metal [6,7,8]. Moreover, heavy metals do not degrade but accumulate in the food chain [2] and additionally, some of them produce carcinogenic and toxic effects in humans and animals [2,9]. Heavy metals, such as zinc (Zn) and copper (Cu), play a vital role in organismal metabolism but can be toxic at high concentrations [10,11,12]. Over the last few decades, there has been a significant increase in the discharge of anthropogenic heavy metals into ecosystems worldwide.

Nowadays the approach to determining heavy metals soil pollution is still based on the analysis of the concentrations of metals in the soils and comparison with specific threshold values. This approach, however, does not provide indications of their deleterious effects on biota [1,2]. On the other hand, another important aspect to studying the toxicity of heavy metals in soil ecosystems is the evaluation of bioavailability and the interactive effects (synergism and/or antagonism) with other pollutants or soil components [13]. In ecotoxicology, biomarkers and bioassays are used to evaluate the toxic action of pollutants in the environment and establish contaminant bioavailability. These tests use bioindicator organisms that are ecologically relevant [1,2]. Invertebrates may serve as effective bioindicators of soil pollution as they are in direct contact with soil pore water or food, unlike many vertebrates that are indirectly exposed through the food chain [2,5]. The most utilised bioindicator species are collembola [13,14], earthworms [1,2,5] and snails [15,16].

Another class of bioindicator organisms are the single-celled ciliated protists (ciliates), which are recognised for their vital role in ecosystems. These eukaryotic micro-organisms have been found in almost any habitat where there is sufficient water for their survival, including soils [17,18,19]. Ciliate diversity is largely unknown, with only 8000 free-living and epibiotic ciliate species identified to date [19]. Ciliates contribute significantly to ecosystem dynamics by decomposing organic matter and transferring energy to higher trophic levels [20,21,22]. They maintain the balance of the ecosystem and play a crucial role in regulating microbial food webs, and this activity appears stimulating mineralization processes in soils [23]. Soil ciliates also have beneficial effects on plant growth through a mechanism known as the “soil microbial loop” [24]. Ciliates are excellent biological indicators of both chemical pollution, particularly heavy metal pollution, and various other types of environmental disturbance [12,22,25,26,27]. Soil ciliate communities provide rapid and valuable ecosystem information, complementing the data provided by more traditional macroscopic bioindicators [28]. Compared to other microorganisms like bacteria and algae, there are fewer studies on the relationship between ciliates and environmental pollutants. However, ciliates are easy to culture and maintain, making them a practical sentinel organism for bioassays [29,30].

Bioassays are widely regarded as effective tools for the assessment of metal pollution, due to their ability to specifically react and detect the available fractions of metals. Metal ions have been demonstrated to trigger the formation of reactive oxygen species (ROS), including superoxide anion (O^2−^), hydroxyl radicals (OH^−^), hydrogen peroxide (H_2_O_2_) and singlet oxygen (1O_2_), through both normal metabolic activities and external sources [31]. It is notable that high levels of ROS can be generated under conditions of heavy metal exposure, resulting in the overloading of antioxidant mechanisms [12,32]. Ciliates, in particular, experience impacts on their ability to feed and on their growth rates, as well as changes in respiration and oxidative stress due to the generation of high levels of ROS caused by heavy metal pollution [25,33]. However, a survey of the literature revealed no available data on the antioxidant properties of soil-dwelling ciliate protists. To our knowledge, this study is the first to investigate the antioxidant properties of the soil ciliated protist *Rigidohymena tetracirrata* in response to heavy metal stress.

Within the MOSYSS project [34], which assessed the biological quality of agricultural soils in the Marche region (Central Italy) using various bioindicators including ciliates, *R. tetracirrata* was consistently detected across arable sites, demonstrating its adaptation to agroecosystems and its value as a representative of soil biological status. Its ubiquity in the field, together with ease of cultivation, a short generation time of about 17 h, and clear physiological responses to pollutants, makes this ciliate particularly suitable for ecotoxicological applications. These combined features identify *R. tetracirrata* as a promising sentinel organism for soil health evaluation.

On this basis, *R. tetracirrata* was selected as the test organism in this study to evaluate its responses to heavy metal exposure and its potential as a model species for ecotoxicological bioassays and soil health monitoring. The study specifically aimed to determine the acute toxicity of single and binary mixtures of Cu, Zn and Cd after 24 h of exposure, while also assessing the antioxidant response of both extracellular (EC) and intracellular (IC) extracts by measuring total phenolic content and free radical scavenging activity. The obtained results highlight the effectiveness of the antioxidant properties of *R. tetracirrata* in scavenging free radicals and metal ions. It also provides evidence that ciliates may be an excellent source of natural antioxidants. Through this approach, this study explores the suitability of *R. tetracirrata* as a eukaryotic model organism for laboratory-based ecotoxicological testing and highlights its potential as a reliable bioindicator of heavy metal contamination in soils.

## 2. Materials and Methods

### 2.1. Origin and Culture Conditions of the Soil Ciliate R. tetracirrata

The test organism, the soil ciliate *R. tetracirrata*, was isolated during monitoring activities conducted within the framework of the MOSYSS project in agricultural fields of the Marche region, Central Italy [34]. Cultures were maintained in Pringsheim’s Medium (PM) [35] at 18 ± 1 °C in the dark and fed with the green alga *Chlorogonium elongatum* (P.A. Dangeard) Francé 1897 [36]. All experiments were performed using cells in the logarithmic growth phase of the ciliate.

### 2.2. Experimental Setup: Metal Salts (Chemicals), Preliminary Range-Finding, and Determination of Final Exposure Concentrations

For the ecotoxicological assays, analytical-grade pure chemicals (purity ≥ 99%) were used as a source of metal ions: cadmium chloride (anhydrous CdCl_2_), zinc sulphate (ZnSO_4_.7H_2_O) and copper (II) sulphate (CuSO_4_.5H_2_O) from Sigma (Milan, Italy). Stock solutions (0.1 M) were made by dissolving each metal salt in PM (pH 7). Preliminary range-finding tests were conducted using a different set of metal concentrations to identify suitable exposure levels for the final experiments. In these initial assays, 6 to 12 concentrations were tested for each metal. The lowest concentration was chosen to have no observable effect on cell viability, while the highest concentration was set to induce more than a 50% reduction in viability. This approach ensured that the final test concentrations included both sublethal and lethal ranges relevant to ecotoxicological assessments. Consequently, the final working solutions were prepared daily by diluting stock solutions to the following nominal concentrations: 0 to 2.5 mg Cd/L, 0 to 0.7 mg Cu/L, and 0 to 45 mg Zn/L. Trypan Blue (TB) was purchased from Merck (Milan, Italy).

### 2.3. Toxicity Tests with Single Metals (Cu, Zn and Cd)

To analyse the toxicity of heavy metals Cu, Zn and Cd on the selected soil ciliate species *R. tetracirrata*, preliminary toxicity range-finding tests using a different set of metal concentrations, were conducted to determine the range of concentrations to be used in the final tests. For each experiment test, a set of 6–12 different concentrations were tested, arranged so that the lowest concentration would have no effect on cell viability and the highest concentration would produce more than 50% loss of viability. Ecotoxicity assays were performed using specific 3-well depression glass slides. One hundred cells were picked from exponentially growing cultures with a micropipette and inoculated into each well in a final volume of 1 mL of PM containing the selected metal concentration, covered to avoid evaporation and incubated in a humid chamber at the temperature 18 ± 1 °C for 24 h. Ciliates were not fed during the test period. After a 24 h period, the cells were checked for mortality and survivorship under the different test concentration solutions under a stereoscopic microscope at 20–40× magnifications. Ciliates were accounted as dead when missing due to cell burst or when standing still at the bottom of a well, unable to swim even after gentle mechanical stimulation with the tip of a micropipette. Furthermore, viability tests using the Trypan Blue (TB) exclusion tests were realised according to Strober [37]. As a control, the same number of ciliates was inoculated into wells containing 1 mL of PM. Replicates (n = 3) for each concentration treatment were averaged. The endpoints utilised for cytotoxicity assessment were the lethal concentration (LC) LC_20_ (concentration causing mortality in 20% of the cells) and LC_50_ (concentration causing mortality in 50% of the cells) at 24 h. Mean mortality values were used to derive a regression equation.

### 2.4. Cytotoxicity Bioassays with Bimetallic Mixtures (Cd + Zn, Cu + Zn, and Cd + Cu)

The bimetallic cytotoxicity bioassays were designed according to Gallego [38] with slight modifications. This procedure is based on the Concentration Addition (CA) model that uses the individual metal toxicities (LCX) and the Toxic Unit (TU) concept [39]. The dimensionless TU represents the relative contribution to the toxicity of the individual chemical in a mixture of chemicals. In this study, the bimetallic mixtures were prepared by combining the toxicants according to their individual LC_50_ values. The effects of three different bimetallic mixtures were then analysed (Cd + Zn, Cu + Zn, and Cd + Cu). 1TU corresponds to the LC_50_ values of single-metal toxicities. The total TU of the binary mixture was the sum of its individual metal fractions. In this experiment, we selected and tested four different concentrations of TU (0.5, 0.75, 1, and 1.25 TUs) to cover a wide range of metal concentration mixtures. The expected mortality rates were calculated as the sum of the single-metal toxicities obtained for each metal in the mixture, at the same concentration. If the observed mortality rate is higher than that of the expected mortality rate, the interaction is synergistic. At the same time, if the observed mortality is lower than the expected death rate, the interaction is antagonism. Cytotoxicity with bimetallic mixtures was performed using the same test method as defined above for the single-metal treatment assay.

### 2.5. Total Phenolic Content and Antioxidant Activity Assays

Three different types of antioxidant assays, namely Total Phenol Content (TPC), α,α-diphenyl-β-picrylhydrazyl (DPPH), and Hydroxyl Radical Scavenging Assay (HRSA), were applied to evaluate the presence of antioxidant activity in *R. tetracirrata* exposed to different concentration of the single and bimetallic mixture of heavy metals. To analyse, the antioxidant activity of *R. tetracirrata* cells (2000 cells/mL) from an exponentially growing culture were transferred into Petri plates in the volume of 10 mL and exposed to different concentrations of single and bimetallic mixtures of heavy metals for 24 h. In single-metal exposure, the LC_20_ and LC_50_ concentrations were used to analyse the antioxidant activity. To evaluate the antioxidant responses under bimetallic mixture, eight concentrations of Cd + Zn mixtures were prepared based on toxic unit (TU) combinations, namely 0.25 + 0.25, 0.5 + 0.25, 0.75 + 0.25, 0.5 + 0.5, 0.25 + 0.5, 1 + 0.25, 0.75 + 0.5, and 0.25 + 1 TUs (Cd + Zn). These specific combinations were selected to explore the interactive effects of Cd and Zn in a bimetallic mixture.

Total phenolic content and antioxidant activities were measured in both the extracellular and intracellular fractions of the cell culture. After 24 h exposure to heavy metals, the ciliates were separated from the medium by gentle centrifugation (4000 rpm for 10–15 min). The cells pellet was then washed in distilled water and resuspended in 1 mL of 50 mM phosphate buffer (pH 7.0). The cells were then homogenised using a Teflon homogeniser for 4–5 min and resuspended in 1 mL of 50 mM phosphate buffer (pH 7.0). The homogenate was then centrifuged at 4000 rpm for 30 min and the resulting pellet was collected. This pellet was termed ‘intracellular’. The extracellular fraction was obtained from the ciliate cell culture. Both extracellular and intra-cellular samples were stored at −20 °C until analysis. All the antioxidant assays were performed according to the methods by Ravindran et al. [40].

### 2.6. Estimation of Total Phenolic Content (TPC)

The analysis of TPC was based on the Folin assay of Vattem and Shetty [41]. In this method, 100 μL of ciliates cell extract was added into the test tube, mixed with 2 mL of sodium bicarbonate, and incubated for 2 min at 18 ± 2 °C. Then, 100 μL of Folin–Ciocalteu reagent was added and incubated in the dark at 18 ± 2 °C for 30 min. After the incubation period, the absorbance was measured at λ = 725 nm using the plate reading spectrophotometer (FLUOstar Omega, BMG LABTECH, Ortenberg, Germany). Gallic acid 1 mg/mL was used as standard and standard curves were obtained using various concentrations of gallic acid. We also used the culture medium (without cells) as a control, as no phenols were observed.

### 2.7. DPPH Scavenging Assay

We used the DPPH scavenging assay to assess the antioxidant capability in the ciliate extracts. Antioxidants activity was determined by its ability to scavenge 1, l-diphenyl-2-picrylhydrazyl (DPPH) radicals. This analysis is based on the procedure of Yıldırım et al. [42]. Briefly, 1 mM DPPH stock solution was prepared in the 95% of ethanol. 800 μL of 1 mM DPPH solution was added to 200 μL of cell extract. The samples were mixed well and incubated in the dark for 30 min at room temperature. After incubation, the samples were transferred to centrifuge tubes and centrifuged at 14,000 rpm for 5 min. Then, the supernatant was collected and the absorbance measured at λ = 517 nm using a spectrophotometer (FLUOstar Omega, BMG LABTECH, Ortenberg, Germany). 200 μL of 95% ethanol was used as a control. Butylated Hydroxy Anisole (BHA) was used as reference compounds. Antioxidant activity is expressed as a percentage (%). The percentage of DPPH scavenging was calculated using the formula below:

$$DPPH\;scavenging\;(\%)\;=\;\frac{control\;absorbance\;-\;extract\;absorbance}{control\;absorbance}\;\times 100$$

### 2.8. Hydroxyl Radical Scavenging Assay (HRSA)

HRSA was evaluated based on the Fenton reaction [43] and followed the procedure of Ravindran et al. [39] with slight modification. The hydroxyl radical is produced by the Fe^3+^ ascorbate EDTA H_2_O_2_ system, i.e., Fenton reaction. To analysis, this reaction, the chemicals of 2–deoxyribose (2.8 mM), FeCl_3_ (100 µM), H_2_O_2_ (1 mM), and EDTA (100 µM) were dissolved in the 20 mM phosphate buffer at pH 7.4. From this chemical mixture, 800 µL was transferred to the test tube. Then 10 µL of ascorbic acid (10 mM) and 100 µL sample extract was added. The ascorbic acid was added and incubated at 37 °C for 1 h. After the incubation, 1 mL of 2.8% TCA, and 1 mL of 1% aqueous TBA were added, and the mixture was heated at 90 °C for 15 min to develop colour. After cooling the absorbance was measured at 532 nm against a suitable blank solution. Mannitol was used as a reference compound. The percentage of scavenging was determined using the formula below.

$$Hydroxyl\;radical\;scavenging\;(\%)=1-\frac{Sample\;absorbance}{Blank\;absorbance}\times 100$$

### 2.9. Statistical Analysis

Statistical analysis was performed using the InfoStat software ver. 2012 and Microsoft^®^ Excel (v.2010); *p* ≤ 0.05 was regarded as statistically significant. The LC_20_ and LC_50_ values were determined using logit-log regression analysis. Concentration data were log-transformed, and mortality proportions were subjected to logit transformation. Linear regression was fitted to the transformed data, and LC values were interpolated from the regression equation. Standard errors and 95% confidence intervals were calculated using the delta method to account for parameter uncertainty. All statistical analyses ensured robust estimation of lethal concentrations and their precision.

For comparisons between heavy metal treatment groups and the control, data were first tested for compliance with parametric assumptions: normality was assessed using the Shapiro–Wilk test, and homogeneity of variances was examined using Levene’s test. As both assumptions were satisfied, comparisons were conducted using a one-way analysis of variance (ANOVA). *Post hoc* comparisons between each treatment group and the control were carried out with Dunnett’s test to adjust for multiple comparisons. Results are presented as the mean ± standard error (SE) of at least three independent replicates.

## 3. Results

### 3.1. Cytotoxicity of Single Metals (Cu, Zn and Cd)

**Figure 1:**
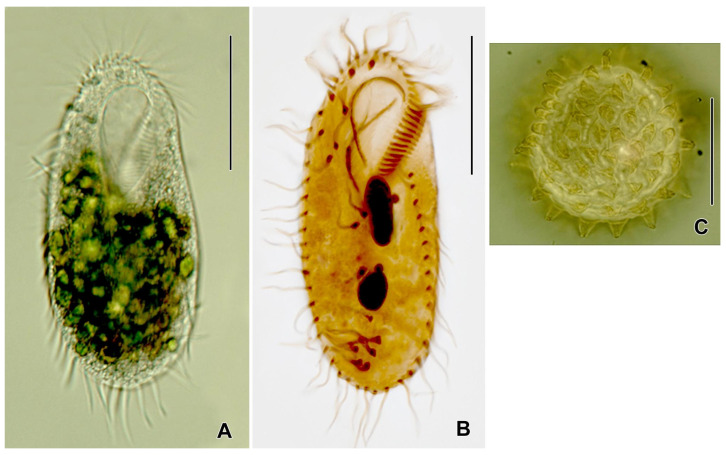
(**A**) *Rigidohymena tetracirrata* from life. (**B**) After protargol impregnation. (**C**) A resting cyst. Scale bars: 40 μm (**A**,**B**), 20 μm (**C**).

In this study, we analysed the toxicity of three heavy metals (Cu, Zn and Cd) on *R. tetracirrata* (Figure 1). The lethal concentrations (LC_20_ and LC_50_) of Cu, Zn and Cd for *R. tetracirrata* were determined using a logit-log regression model. The calculated values, along with their standard errors and 95% confidence intervals, are presented in Table 1. The model demonstrated an excellent fit to the experimental data for all metals, as indicated by high R^2^ values (0.961–0.985). The results indicate that *R. tetracirrata* exhibits the highest degree of resistance to Zn. The cellular toxicity of Cu was higher than that of Zn or Cd. The most sensitive effect of heavy metals on *R. tetracirrata* was observed with Cu. However, in comparison to Cu, the ciliate exhibits a heightened susceptibility to the cytotoxic effects of Cd and Zn. The LC_20_ and LC_50_ values for Cd are 0.68 and 1.12 mg L^−1^, and for Zn are 19.86 and 44.12 mg L^−1^, respectively (Table 1). The LC_20_ and LC_50_ values for Cu are 0.16 and 0.25 mg L^−1^ (Table 1), respectively. These findings clearly demonstrate that Cu is the most toxic of the three metals tested in this ciliate species. The decreasing order of toxicity is as follows: Cu > Cd > Zn. The narrow confidence intervals for all estimates indicate a high degree of precision and reliability in the determined lethal concentrations.

### 3.2. Cytotoxicity of Bimetallic Mixtures (Cd + Zn, Cu + Zn and Cd + Cu)

**Figure 2:**
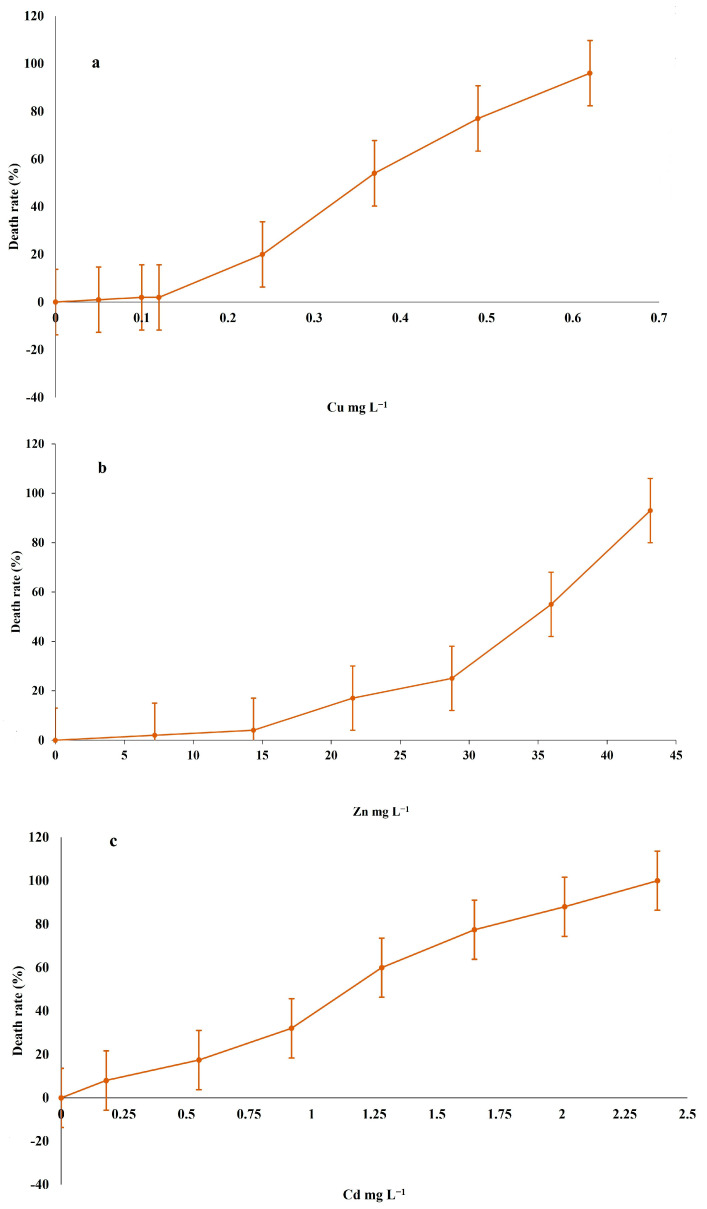
Death rate percentages (each point represents a mean value ± standard deviation) obtained from populations of *R. tetracirrata*, after 24 h exposure at different heavy metal concentrations (mg L^−1^). (**a**) Cu, (**b**) Zn and (**c**) Cd treatments.

In the analyses of bimetallic mixtures, three types of mixtures (Cd + Zn, Cu + Zn, and Cd + Cu) were used. The calculation of the expected level of cytotoxicity was performed based on the percentage graph of the death rate. (Figure 2a–c).

Our study shows that exposure to bimetallic mixtures of Cd + Zn, resulted in a greater degree of antagonistic effects (see Table 2) compared to other bimetallic mixtures. The ciliate exhibited antagonistic activity under concentrations of 0.25 + 0.25, 0.5 + 0.25, 0.75 + 0.25, 0.5 + 0.5, 0.25 + 0.75, and 1 + 0.25 (Cd + Zn). However, at higher concentrations exceeding 1 TU (Cd + Zn), the bimetallic mixture interaction changed based on the impact of each metal’s cytotoxicity. For instance, at 0.75 + 0.5 TU (Cd + Zn), the expected value for the median effective concentration (EC_50_) was 34, but the observed value was 45.83 ± 3.87, indicating a synergistic effect. Conversely at concentrations of 1 + 0.25 TU, an antagonism effect was observed, suggesting that 1 TU of Cd metal was less toxic when mixed with 0.25 TU of Zn metal (Table 2).

The remaining two sets of bimetallic mixtures, Cu + Zn and Cd + Cu, showed similar outcomes with minimal variation, predominantly yielding effects that were not considerably disparate. Cu + Zn bimetallic mixtures exhibited synergistic effects at three different TU concentrations, influenced by the higher impact of Zn compared to Cu (Table 3).

Furthermore Cd + Cu mixtures also showed synergistic interactions, particularly at concentrations 0.75 + 0.25, 0.5 + 0.5, 0.25 + 0.75 and 0.25 + 1.0 TU, while antagonistic interactions at concentrations 0.5 + 0.25 and 0.75 + 0.5 TU. In these mixtures, Cd metal was found to be less toxic to *R. tetracirrata* than Cu. However, at 1 TU exposure, the interaction became synergistic, indicating that the impact of Cu was higher than that of Cd in terms of its cytotoxic effect (Table 4).

### 3.3. Antioxidant Properties of R. tetracirrata Extracts Treated with Cu, Zn, Cd, and Cd +Zn

#### 3.3.1. Total Phenolic Content (TPC) from Extracts of *R. tetracirrata*

**Figure 3:**
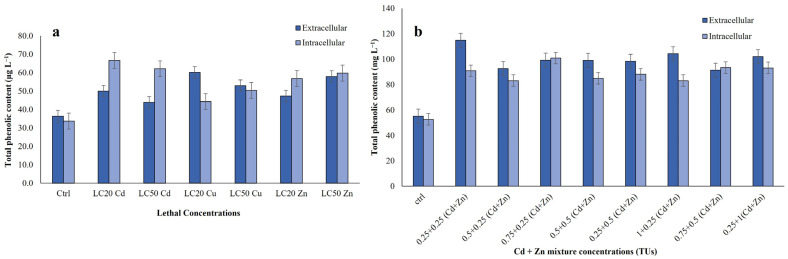
Total phenolic content measured in the *R. tetracirrata* cell extracts (**a**) the exposure to the single-metal (Cu, Zn and Cd) concentrations corresponding to the LC_20_ and LC_50_ values (**b**) the exposure to the bimetallic mixture concentrations (0.25 + 0.25, 0.5 + 0.25, 0.75 + 0.25, 0.5 + 0.5, 0.25 + 0.5, 1 + 0.25, 0.75 + 0.5, and 0.25 + 1 TUs).

The total phenolic content of ciliate cell extracts exposed to single (Cu, Zn and Cd) and bimetallic mixture (Cd + Zn) was analysed (Figure 3a,b). The highest TPC value of ciliate cell extracts was observed with Cu LC_20_ value of extracellular and Cd LC_20_ value of intracellular (*p* ≤ 0.01) (Figure 3a). Under single-metal treatments, intracellular TPC was generally higher than that of extracellular content, except in Cu-exposed cells. In contrast, bimetallic treatments showed the opposite trend, with extracellular TPC exceeding intracellular values. The TPC of ciliate cell extracts was highest with 0.25 + 0.25 TU (Cd + Zn) of extracellular and 0.75 + 0.25 TU (Cd + Zn) for intracellular (*p* ≤ 0.001) (Figure 3b).

Parametric assumptions were met for both intracellular and extracellular data (Shapiro–Wilk *p* > 0.05; Levene’s *p* > 0.05). One-way ANOVA indicated a significant treatment effect on intracellular (F = 700.81, *p* < 0.001, η^2^ = 0.997) and extracellular TPC (F = 454.27, *p* < 0.001, η^2^ = 0.995). Dunnett’s *post hoc* tests confirmed that all metal exposures significantly differed from the control (*p* < 0.001; Table S1). Intracellular TPC was significantly reduced across all treatments, with the strongest suppression under Cd (−32.1% at LC_20_, −27.8% at LC_50_), followed by Zn (−22.9% at LC_20_, −25.3% at LC_50_) and Cu (−11.1% at LC_20_, −16.4% at LC_50_). Conversely, extracellular TPC was significantly elevated. The largest increases occurred under Cu (mean difference from control (Δ) +23.5 at LC_20_, +16.5 at LC_50_) and Zn (Δ +11.4 at LC_20_, +21.6 at LC_50_), while Cd induced smaller yet consistent increases (Δ +12.6 at LC_20_, +6.7 at LC_50_).

Cd + Zn mixtures also met parametric assumptions (Shapiro–Wilk *p* > 0.05; homogeneous variances). One-way ANOVA revealed highly significant treatment effects on intracellular (IC: F = 1865.0, *p* < 0.0001) and extracellular (EC: F = 6150.3, *p* < 0.0001) TPC. All mixtures significantly differed from the control (Dunnett’s test, *p* < 0.05). Intracellular TPC peaked at the IC 0.75 + 0.25 mixture (100.96) but declined at higher Cd concentrations (e.g., IC 1 + 0.25: 83.50). Extracellular TPC was consistently elevated, reaching a maximum at EC 0.25 + 0.25 (115.12) and a minimum at EC 0.75 + 0.5 (91.96) (Table S2).

Notably, both single-metal and Cd + Zn treatments significantly increased total phenolic and antioxidant levels compared to untreated controls (*p* ≤ 0.01 and *p* ≤ 0.001, respectively; Figure 3a,b). While single-metal exposures induced phenolic release at the µg L^−1^ scale, bimetallic mixtures triggered release at the mg L^−1^ scale, with TPC levels in Cd + Zn treated extracts being approximately 103 fold higher than in single-metal treatments.

#### 3.3.2. DPPH Scavenging Activity from Extracts of *R. tetracirrata*

**Figure 4:**
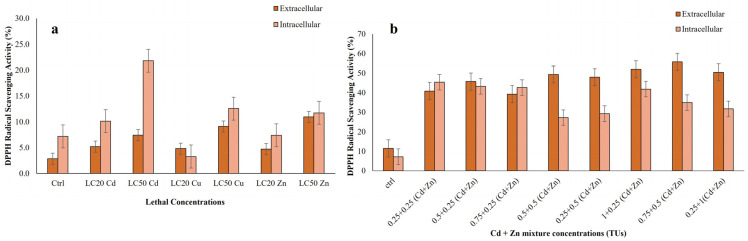
DPPH scavenging activities measured in the *R. tetracirrata* cell extracts (**a**) the exposure to the single-metal (Cu, Zn and Cd) concentrations corresponding to the LC_20_ and LC_50_ values (**b**) the exposure to the bimetallic mixture concentrations (0.25 + 0.25, 0.5 + 0.25, 0.75 + 0.25, 0.5 + 0.5, 0.25 + 0.5, 1 + 0.25, 0.75 + 0.5, and 0.25 + 1 TUs).

The free radical scavenging activities of ciliate cell extracts was determined by the DPPH assay. Extracts exhibited a clear concentration-dependent antiradical effect, as indicated by the inhibition of DPPH radicals (Figure 4a,b). In single-metal treatments, the Zn LC_50_ and Cd LC_50_ values in intracellular and extracellular contents exhibited a higher DPPH scavenging activity, with a significant value of *p* ≤ 0.05 (Figure 4a). In summary, DPPH scavenging activity in single-metal-treated ciliate extracts was found to be higher in extracellular than intracellular contents.

Data for both compartments met the assumptions of normality (Shapiro–Wilk *p* > 0.05) and homoscedasticity (Levene’s *p* > 0.05). One-way ANOVA revealed highly significant differences among treatments for intracellular (F = 264.80, *p* < 0.001, η^2^ = 0.99) and extracellular (F = 130.43, *p* < 0.001, η^2^ = 0.98) activities. All metal treatments significantly differed from the control (Dunnett’s test, *p* < 0.05; Table S3). Intracellular activity increased most markedly under Cd (22.12 at LC_50_ vs. control 7.07), followed by Cu (13.03 at LC_50_) and Zn (12.06 at LC_50_). Extracellularly, Zn elicited the strongest response (11.09 at LC_50_ vs. control 3.07), followed by Cu (9.74) and Cd (7.79). Effect sizes (Cohen’s d) were very large across all treatments, indicating that extracellular antioxidant activity was more responsive than intracellular defence.

Our results demonstrated that both single metals (Cu, Zn and Cd) and bimetallic mixtures (Cd + Zn) exhibited scavenging activity; with bimetallic mixtures (Cd + Zn) showing more pronounced DPPH activities. The maximal activity was observed with 0.25 + 0.25 TU (Cd + Zn) in intracellular extract and 0.75 + 0.5 TU (Cd + Zn) in extracellular extract (*p* ≤ 0.001) (Figure 4b). Data assumptions for ANOVA were met. The analysis showed highly significant treatment effects for both intracellular (F = 3842.06, *p* < 0.001, η^2^ = 0.999) and extracellular (F = 6265.62, *p* < 0.001, η^2^ = 0.999) assays, with all Cd + Zn mixtures differing significantly from the control (Dunnett’s test, *p* < 0.05). Intracellular scavenging activity increased sharply from a control value of 7.49, reaching a maximum of 45.68 (IC 0.25 + 0.25). Higher-concentration combinations like IC 0.5 + 0.5 and IC 0.25 + 1 showed reduced activity (27.63 and 31.77, respectively). In contrast, extracellular activity rose consistently from a control value of 11.74, peaking at 55.73 (EC 0.75 + 0.5) (Table S4). Extremely large Cohen’s d values confirmed robust metal-induced responses.

In summary, exposure to heavy metals significantly enhanced the antioxidant capacity of *R. tetracirrata*, with bimetallic Cd + Zn mixtures inducing the strongest scavenging activity compared to both single-metal treatments (*p* ≤ 0.05) and untreated controls.

#### 3.3.3. Hydroxyl Radical Scavenging Activity of Extracts of *R. tetracirrata*

**Figure 5:**
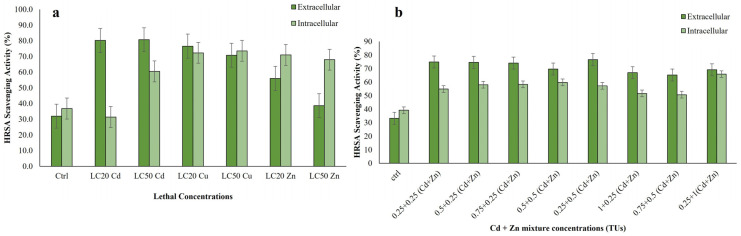
Hydroxyl radical scavenging activities measured in the *R. tetracirrata* cell extracts (**a**) the exposure to the single-metal (Cu, Zn and Cd) concentrations corresponding to the LC_20_ and LC_50_ values (**b**) the exposure to the bimetallic mixture concentrations (0.25 + 0.25, 0.5 + 0.25, 0.75 + 0.25, 0.5 + 0.5, 0.25 + 0.5, 1 + 0.25, 0.75 + 0.5, and 0.25 + 1 TUs).

The hydroxyl radical scavenging activity (HRSA) of ciliate extracts increased in response to both single and bimetallic metal exposures, with effects being concentration dependent (Figure 5a,b). In single-metal treatments, the LC_50_ for the extracellular extract of Cd and the LC_50_ for the intracellular extract of Cu exhibited higher hydroxyl radical scavenging activity (*p* < 0.001) (Figure 5a). The assay shows that ciliate cell extracts possess the capacity to inhibit hydroxyl radical-mediated deoxyribose degradation in a Fe^3+^ EDTA ascorbic acid and H_2_O_2_ reaction mixture. Data for intracellular and extracellular HRSA met the assumptions for parametric testing. One-way ANOVA revealed highly significant metal-induced effects in both compartments (intracellular: F = 3355.03, *p* < 0.0001, η^2^ = 0.999; extracellular: F = 2949.95, *p* < 0.0001, η^2^ = 0.999). The response was metal and concentration dependent. Intracellularly, Cd exhibited a biphasic response, with suppression at LC_20_ but strong enhancement at LC_50_, while Cu and Zn consistently reduced activity. Extracellularly, all metals significantly inhibited HRSA relative to the control, with Cd showing the strongest suppression, followed by Cu and Zn. Effect sizes were very large (Cohen’s d > 9.4) for all significant comparisons (Table S5). Data were normally distributed. While Levene’s test indicated inhomogeneity of variance for the extracellular (EC) data (*p* < 0.001), the robustness of ANOVA was upheld by equal sample sizes (n = 3).

In the bimetallic mixture treatments, the intracellular HRSA was found to be lower in comparison to the extracellular content. Highly significant treatment effects were found for both intracellular (F = 2943.23, *p* < 0.0001, η^2^ = 1.00) and extracellular (F = 34.48, *p* < 0.0001, η^2^ = 0.94) assays. All Cd + Zn mixtures significantly increased HRSA compared to the control (*p* < 0.001). Intracellular activity was highest for IC 0.25 + 1 (65.99) and lowest for IC 0.75 + 0.5 (51.00). Extracellular activity was greatest for EC 0.25 + 0.5 (76.79) and lowest for EC 0.75 + 0.5 (65.51). All treatments produced very large effect sizes (Cohen’s d > 0.8) (Table S6).

Overall, the strongest HRSA responses were observed in extracellular extracts at 0.25 + 0.25 TU (Cd + Zn) and in intracellular extracts at 0.25 + 1 TU (Cd + Zn) (*p* ≤ 0.001). Both single and bimetallic metal exposures significantly enhanced antioxidant capacity relative to untreated controls (*p* ≤ 0.001; Figure 5b).

### 3.4. A Brief Note on the Genus Rigidohymena and Its Potential as a Bioindicator of Soil Health

The genus *Rigidohymena* [44] currently comprises three species: *R. tetracirrata* [44,45]; *R. quadrinucleata* [44,46]; and *R. candens* [44,47]. Species of the genus are characterised by a large oral apparatus, i.e., *Cyrtohymena* pattern [48,49], which facilitates its feeding on wide range of microorganisms, e.g., amoeba, flagellates, fungal spores, and even small ciliates. They inhabit both freshwater and terrestrial habitats. Combined with its widespread occurrence in agricultural soils, these features make *R. tetracirrata* an ideal candidate for ecotoxicological studies. Similarly to other members of the subfamily Stylonychinae [50], such as *Stylonychia* and *Tetmemena*, which have been extensively employed in ecotoxicology [48,51,52].

Detailed morphological information is available for the type species *R. tetracirrata*, which was originally described as *Cyrtohymena tetracirrata* [45,53,54] and later transferred to *Rigidohymena* by Berger [44]. This reassignment was based on its rigid body, relatively long adoral zone, and the posteriorly displaced cirrus V/3, which clearly indicates that it is not involved in primordium formation [44,48]. A more detailed account of the morphology, ontogenesis, and molecular phylogeny (SSU rRNA gene sequence) of the Italian *R. tetracirrata* population will be provided in a forthcoming study (Bharti, Kumar & La Terza, manuscript in preparation).

## 4. Discussion

The majority of ecotoxicological studies on heavy metal cytotoxicity have focused on freshwater protozoa, those isolated from urban wastewater treatment plants, or standard ciliates like *Tetrahymena* spp. [25,55,56]. To date, there has been a paucity of research on the toxic effects of heavy metals specifically on soil ciliates [57,58]. In the present study, we have evaluated the cytotoxicity of Cu, Zn and Cd on a population of the ciliate *R. tetracirrata*. The outcome of our study indicates that the observed toxicity sequence of metals used is Cu > Cd > Zn. The median lethal concentration (LC_50_) values for Cu, Zn and Cd heavy metals were estimated to be 0.25, 44.12, and 1.12 mg L^−1^, respectively. Furthermore *R. tetracirrata* cells appear to be more resistance to Zn, Cd and Cu than other freshwater ciliate species studied to date [56,58]. This finding agrees with the observations reported by Madoni and Romeo [59], who found that the freshwater ciliate *Colpidium colpoda* exhibited higher toxicity to Cu than to Cd. This finding is consistent with the observations of Díaz et al. [57] and Martín-González [60], who found that the order of toxicity was Cd > Cu > Zn in ciliated protists isolated from soils and urban wastewater treatment plants. However, in comparison with the data presented here, these values and the toxicity order rank are lower. On the other hand, the ciliate *T. pyriformis* is more resistant to these metals [59,60]. Echavez and Leal [61] isolated some ciliates from Lake Maracaibo and discovered that *Euplotes* sp. (18.5 mg L^−1^), *Oxytricha* sp. (9.2 mg L^−1^), *Coleps* sp. (53.3 mg L^−1^), and *Chilodonella* sp. (19.2 mgL^−1^) were more resistant to Cd after a one-hour exposure. Meanwhile, Marín-Leal et al. [62] isolated some ciliates from the same lake and found that *Uronema* sp. (0.71 mg L^−1^), *Euplotes* sp. (0.72 mg L^−1^), and *Loxodes* sp. (0.14 mg L^−1^) were more sensitive to Cd after a one-hour exposure. The LC_50_ values for these species were found to exceed the values obtained in this study. It is important to note that the cited data were obtained from one-hour exposures, whereas the present study employed a 24 h exposure period. Differences in exposure duration can significantly influence toxicity outcomes, as longer exposures often result in lower LC values due to cumulative stress on the organisms. This distinction is important for accurate comparison of toxicity thresholds across studies. Gallego et al. [38] reported that the LC_50_ for Cu, Zn, and Cd in the ciliate *T. thermophila* were 0.47, 3.58, and 0.195 mg L^−1^, respectively. These values are lower than those obtained in the present study. Consequently, it can be concluded that our strain of *R. tetracirrata* generally exhibits increased sensitivity to Zn, Cd, and Cu in comparison with other ciliated protists.

In the context of bimetallic mixtures, Cd + Zn exhibited a greater degree of antagonistic activity in comparison to other metallic mixtures including Cu + Zn and Cd + Cu. Furthermore *R. tetracirrata* showed that both metallic components within the mixture were significant contributors to biotoxicity. In *R. tetracirrata*, the combined effects of Cd + Zn at 0.5 to 1 TUs were antagonistic, but under high concentrations of heavy metal (above 1 TUs) the interaction changed to synergistic. Most studies have shown that the toxicity of Cd is reduced in the presence of Zn [33,38,63,64,65,66]. However, some studies have reported that the presence of low or moderate Zn concentrations, enhances Cd cytotoxicity in ciliated protists [57,60]. In contrast to the findings of numerous studies showing a decrease in Cd toxicity in the presence of Zn, our observations indicate that *R. tetracirrata* exhibits increased resistance to Cd when simultaneously exposed to Zn. In this soil ciliate, the presence of low and moderate concentrations of Zn reduces the biological toxic effect of Cd. This phenomenon has been observed in other organisms, including *T. pyriformis*, *T. thermophila*, *Daphnia magna* and other invertebrates [63].

The combined toxic effects of Cu + Zn and Cd + Cu could be considered either antagonistic or synergistic, depending on the toxicity units (TUs) tested in *R. tetracirrata*. The combined toxic effects of Cu + Zn and Cd + Cu could be considered either antagonistic or synergistic, depending on the toxicity units (TUs) tested in *R. tetracirrata*. The potential for these interactive effects (i.e., antagonism, synergism) to vary over time was not addressed in the present study but represents a critical avenue for future research. It was observed that both metallic mixtures (Cu + Zn and Cd + Cu) exhibited similar behaviour, with cases of antagonism, synergism, and, in most concentrations, no significant differences (Table 3 and Table 4). In the Cu + Zn bimetallic mixture, the type of interaction shifts from antagonism to synergism with the addition of Zn. Concurrently, the toxicity exhibited by the Cu + Zn mixture was found to exceed that induced by Cu alone. *R. tetracirrata* demonstrates variations from antagonism to synergism, depending on the metal concentration. Consistent with these observations, analogous results have been reported in other organisms including the ciliate *T. thermophila* [38], bacteria [64], microalgae [66], crustaceans [65], insects [63], and plants [67].

However, the antagonism of Cu + Zn, remains ambiguous, and it is conceivable that Cu bioaccumulation is not a substantial process in this ciliate species. In the context of heavy metal toxicity, such as elevated Cu levels within cells, the formation of reactive oxygen species (ROS) has been observed, potentially resulting in damage to lipids, nucleic acids, and proteins [68]. In Cd + Cu interaction, analogous activities have been observed, though it is generally regarded as a more pronounced effect than simple addition [69]. This type of interaction in the binary metal combinations of Cd + Cu has been observed by Gallego [38] in the ciliate *T. thermophila*. While our study emphasised endpoint-specific interactions, we acknowledge that a broader statistical perspective such as a non-parametric test evaluating whether antagonism generally predominates across the dataset could provide valuable complementary insights. We suggest this approach as a promising direction for future research.

This study also analysed the total phenolic content (TPC) and antioxidant activity of this soil ciliate species [33]. Specifically, the TPC and antioxidant activity of cell extracts from *R. tetracirrata* were examined, confirming the presence of phenolic compounds and antioxidant properties. Phenols, as primary chemical elements, are responsible for reducing lipid peroxidation and thus function as both primary and secondary antioxidants [70]. Furthermore, it was observed that cell extracts with high phenolic content also exhibited notable antioxidant activity. Significant differences between total phenol content and antioxidant activity were detected using one-way ANOVA followed by Dunnett’s test (*p* ≤ 0.05), suggesting that phenols play an important role in ciliates through their hydroxyl group mediated free radical scavenging ability. Consequently, the phenolic content of ciliates may directly contribute to their antioxidant activity. Statistical analysis (one-way ANOVA and Dunnett’s test) showed that heavy metal stress significantly altered the TPC of *R. tetracirrata*. Cd strongly reduced intracellular TPC, while Cu induced pronounced extracellular release. In contrast, Cd + Zn mixtures enhanced overall phenolic production, with extracellular levels consistently higher than intracellular, indicating active exudation for detoxification. The ciliate cell extract in this study demonstrated a higher than antioxidant capacity, attributed to its elevated phenolic content, particularly 66.7 mg L^−1^ in single-metal treatments and 114.9 mg L^−1^ in binary metallic mixtures. These values exceed those previously reported in studies on other organisms. For instance, Ravindran et al. [40] reported excellent antioxidant capacity in a halophilic pathogenic fungus (NIOCC 1) and the endophytic fungi *Nerium oleander L.* and liverwort *Scapania verrucosa*.

The findings of this study demonstrate that the soil-dwelling ciliate *R. tetracirrata* has antioxidant activity, indicating its potential as a natural antioxidant. This study is the first to investigate and report the total phenolic content (TPC) and antioxidant activity of *R. tetracirrata* cell extract and in general for protozoan ciliates. Since many of the widely recognised strong synthetic antioxidants often contain carcinogenic compounds, the search for potential antioxidants is crucial, particularly in the context of soil ciliates. Phenolic compounds, which are important constituents of plant and fungi, are valued for their hydroxyl groups, which confer scavenging ability [71]. Consequently, these compounds may play an important role in ciliates. In this study, we therefore set out to determine the total phenolic content of ciliate cell extracts, with a view to evaluating their antioxidant activity against different concentrations of heavy metal stresses using various antioxidant assays.

Two different assays were employed in this study, with the DPPH free radical scavenging assay being a basic and widely used method. This assay is widely regarded as the most accurate screening method for evaluating antioxidant activity, using DPPH as the substrate. Cysteine, glutathione, ascorbic acid, tocopherol, polyhydroxy aromatic compounds (e.g., hydroquinone, pyrogallol, gallic acid), and aromatic amines (e.g., p-phenylene diamine, p-aminophenol), have been found to reduce and decolorise α,α-diphenyl-β-picrylhydrazyl through their hydrogen-donating ability. Previous studies, including Ravindran et al. [40] on the marine-derived pathogenic fungus (NIOCC 1), reported DPPH scavenging activity. Further, Uddin et al. [72] reported DPPH antioxidant activity in the plants *Bergeniacaliata*, and Horta et al. [73] reported in the bacteria *Bifurcaria bifurcata*. The results of the ciliate cell extracts in this study demonstrate their hydrogen-donating capabilities, acting as antioxidants. Statistical analysis revealed that both intracellular and extracellular DPPH activity increased in a metal specific manner. Cd strongly induced intracellular activity, suggesting enhanced internal defence, whereas Zn triggered the highest extracellular response, likely reflecting exudation of metal-chelating compounds to limit oxidative stress. Under Cd + Zn co-exposure, intracellular activity declined in highly imbalanced mixtures, while extracellular activity increased dose-dependently, indicating a shift toward exuded radical scavengers as a first line of defence. Our experimental findings indicate that the soil ciliate *R. tetracirrata* produces a higher level of antioxidants under conditions of heavy metals stress, employing both intracellular and extracellular detoxification strategies tailored to the specific oxidative challenges.

Hydrogen peroxide itself is not very reactive, but it has been observed to decompose rapidly into oxygen and water, thereby producing hydroxyl radicals (OH^−^). These radicals have been demonstrated to cause DNA damage [74]. Hydroxyl radicals are the primary active oxygen species responsible for lipid peroxidation and significant biological damage. In this study, hydroxyl radicals were generated by incubating ferric-EDTA with ascorbic acid and H_2_O_2_ at pH 7.4, and these radicals reacted with 2-deoxy-2-ribose to generate a malondialdehyde (MDA)-like product. This compound forms a pink chromogen upon heating with TBA at low pH. When ciliate cell extracts were added to the reaction mixture, they were found to remove the hydroxyl radicals from the sugar, thereby preventing the reaction. This outcome is consistent with the observations reported by Ravindran et al. [40] and Sharma et al. [75] in their studies of the free radical hydroxyl activity of the green hull of *Juglans regia*. Statistical analysis showed that Cd produced a biphasic effect on HRSA, Cu and Zn suppressed intracellular activity, while Cd + Zn mixtures enhanced both intracellular and extracellular HRSA, particularly at moderate concentrations. These patterns indicate that *R. tetracirrata* employs compartment-specific antioxidant strategies under heavy metal stress. Consequently, the in vitro study clearly demonstrated that the antioxidants present in the soil ciliate *R. tetracirrata* can inhibit the process of metal ion-dependent hydroxyl radical formation and mitigate hydroxyl radical induced cell damage under heavy metals stress conditions. Under such conditions, the role of antioxidant enzymes in cells is also significant. Numerous studies have documented the presence of antioxidant enzymes (Superoxide dismutase, Catalase, Glutathione peroxidase and reductase) and stress genes (Heat shock proteins and Metallothionein) in ciliate species of the genera *Tetrahymena* and *Euplotes* [25,76,77,78].

## 5. Conclusions

The soil ciliate *R. tetracirrata* demonstrates strong potential as a model organism in ecotoxicological research and as a sentinel species for assessing heavy metal contamination in soils. Exposure to different metal concentrations induced the marked production of antioxidant compounds and enhanced radical-scavenging activities, highlighting its dual role as both a sensitive bioindicator and a natural source of antioxidant metabolites. These findings establish *R. tetracirrata* as a valuable tool for monitoring soil health under metal stress and for exploring microbial contributions to natural antioxidant reservoirs. Future work will focus on characterising antioxidant enzyme activities and profiling stress-related gene expression to further elucidate the molecular basis of its defence responses.

## Figures and Tables

**Table 1:** Lethal concentration estimates (LC_20_ and LC_50_) for Cu, Zn and Cd in *R. tetracirrata* derived from logit-log regression analysis, with 95% Confidence Intervals (CI).

S. No:	HMs	Parameter	Estimate (±SE)	95% CI	R^2^
1	Cu	LC_20_	0.16 ± 0.03	0.11–0.23	0.974
		LC_50_	0.25 ± 0.04	0.18–0.38	
2	Zn	LC_20_	19.86 ± 3.25	14.32–27.54	0.961
		LC_50_	44.12 ± 6.18	32.18–58.66	
3	Cd	LC_20_	0.68 ± 0.09	0.52–0.89	0.985
		LC_50_	1.12 ± 0.12	0.85–1.48	

Note: concentrations are in mg L^−1^.

**Table 2:** Observed and predicted cytotoxicity (% mortality) of *R. tetracirrata* to different mixtures of Cd + Zn after 24 h exposition.

Cd + Zn Total TU ^a^	Concentrations (TU) ^a^ for Each Metal		Obtained Cytotoxicity ^b^	Expected Cytotoxicity ^b^	Interaction Type
	Cd	Zn			
0.5	0.5	0	12.77 ± 2.54	18 ± 0.04	Not significant different
	0.25	0.25	6.07 ± 2.54	13 ± 0.59	Antagonism
	0	0.5	3.87 ± 0.98	4 ± 1.17	Not significant different
0.75	0.75	0	29.4 ± 3.48	30 ± 0.06	Not significant different
	0.5	0.25	4.97 ± 1.65	20 ± 0.59	Antagonism
	0.25	0.5	18.83 ± 2.54	15 ± 1.17	Not significant different
	0	0.75	16.63 ± 1.65	18 ± 1.76	Not significant different
1	1	0	49.97 ± 1.65	50 ± 0.08	Not significant different
	0.75	0.25	22.2 ± 2.55	32 ± 0.59	Antagonism
	0.5	0.5	3.87 ± 0.98	22 ± 0.59	Antagonism
	0.25	0.75	8.87 ± 0.98	29 ± 1.76	Antagonism
	0	1	49.43 ± 0.98	50 ± 2.34	Not significant different
1.25	1.25	0	61.07 ± 2.54	68 ± 0.1	Not significant different
	1	0.25	33.3 ± 1.7	52 ± 0.59	Antagonism
	0.75	0.5	45.53 ± 3.87	34 ± 1.17	Synergism
	0.5	0.75	32.77 ± 2.54	36 ± 1.76	Not significant different
	0.25	1	14.97 ± 1.65	61 ± 2.34	Antagonism
	0	1.25	77.77 ± 2.54	82 ± 2.93	Not significant different

^a^ Toxic units, mg L^−1^; ^b^ % mortality ± standard deviation.

**Table 3:** Observed and predicted cytotoxicity (% mortality) of *R. tetracirrata* to different mixtures of Cu + Zn after 24 h exposition.

Cu + Zn Total TU ^a^	Concentrations (TU) ^a^ for Each Metal		Obtained Cytotoxicity ^b^	Expected Cytotoxicity ^b^	Interaction Type
	Cu	Zn			
0.5	0.5	0	12.17 ± 1.19	11 ± 0.04	Not significant different
	0.25	0.25	4.43 ± 2.54	4 ± 0.59	Antagonism
	0	0.5	6.07 ± 0.98	4 ± 1.17	Not significant different
0.75	0.75	0	28.30 ± 3.44	30 ± 0.04	Not significant different
	0.5	0.25	6.07 ± 7.86	13 ± 0.59	Antagonism
	0.25	0.5	9.97 ± 0.92	6 ± 1.17	Synergism
	0	0.75	22.20 ± 2.54	18 ± 1.76	Not significant different
1	1	0	48.83 ± 3.48	50 ± 0.05	Not significant different
	0.75	0.25	34.93 ± 1.65	32 ± 0.59	Not significant different
	0.5	0.5	11.63 ± 2.54	15 ± 0.59	Not significant different
	0.25	0.75	23.87 ± 2.55	20 ± 1.76	Synergism
	0	1	52.20 ± 2.54	50 ± 2.34	Not significant different
1.25	1.25	0	69.40 ± 2.55	71 ± 0.06	Not significant different
	1	0.25	31.63 ± 3.48	52 ± 0.59	Antagonism
	0.75	0.5	28.87 ± 3.81	34 ± 1.17	Not significant different
	0.5	0.75	32.77 ± 2.54	29 ± 1.76	Not significant different
	0.25	1	72.77 ± 1.65	52 ± 2.34	Synergism
	0	1.25	80.53 ± 1.96	82 ± 2.93	Not significant different

^a^ Toxic units, mg L^−1^; ^b^ % mortality ± standard deviation.

**Table 4:** Observed and predicted cytotoxicity (% mortality) of *R. tetracirrata* to different mixtures of Cd + Cu after 24 h exposition.

Cd + Cu Total TU ^a^	Concentrations (TU) ^a^ for Each Metal		Obtained Cytotoxicity ^b^	Expected Cytotoxicity ^b^	Interaction Type
	Cd	Cu			
0.5	0.5	0	16.30 ± 1.91	18 ± 0.04	Not significant different
	0.25	0.25	10.53 ± 2.54	13 ± 0.02	Not significant different
	0	0.5	13.87 ± 0.98	11 ± 0.03	Not significant different
0.75	0.75	0	28.87 ± 3.44	30 ± 0.06	Not significant different
	0.5	0.25	13.87 ± 7.86	20 ± 0.04	Antagonism
	0.25	0.5	20.53 ± 0.92	22 ± 0.03	Not significant different
	0	0.75	32.77 ± 2.54	30 ± 0.04	Not significant different
1	1	0	49.40 ± 3.48	50 ± 0.08	Not significant different
	0.75	0.25	34.97 ± 1.65	32 ± 0.06	Synergism
	0.5	0.5	38.83 ± 2.54	29 ± 0.05	Synergism
	0.25	0.75	44.40 ± 2.55	41 ± 0.04	Synergism
	0	1	48.83 ± 2.54	50 ± 0.05	Not significant different
1.25	1.25	0	72.70 ± 2.55	68 ± 0.10	Not significant different
	1	0.25	55.50 ± 3.54	52 ± 0.08	Not significant different
	0.75	0.5	34.40 ± 3.81	41 ± 0.07	Antagonism
	0.5	0.75	45.53 ± 2.54	48 ± 0.05	Not significant different
	0.25	1	84.97 ± 1.65	61 ± 0.05	Synergism
	0	1.25	73.87 ± 1.96	71 ± 0.06	Not significant different

^a^ Toxic units, mg L^−1^; ^b^ % mortality ± standard deviation.

## Data Availability

The original contributions presented in this study are included in the article/Supplementary Materials. Further inquiries can be directed to the corresponding author.

## Supplementary Materials

The following supporting information can be downloaded at: https://www.mdpi.com/article/10.3390/jox15050169/s1, Table S1: Dunnett’s test summary for intracellular and extracellular TPC in *R. tetracirrata* exposed to single metals compared with control. Values are expressed as mean ± SE; Table S2: Dunnett’s test summary for intracellular and extracellular TPC in *R. tetracirrata* exposed to Cd + Zn bimetallic mixtures compared with control. Values are expressed as mean ± SE; Table S3: Dunnett’s test summary for intracellular and extracellular DPPH in *R. tetracirrata* exposed to single metals compared with control. Values are expressed as mean ± SE; Table S4: Dunnett’s test summary for intracellular and extracellular DPPH in *R. tetracirrata* exposed to Cd + Zn bimetallic mixtures compared with control. Values are expressed as mean ± SE; Table S5: Dunnett’s test summary for intracellular and extracellular HRSA in *R. tetracirrata* exposed to single metals compared with control. Values are expressed as mean ± SE; Table S6: Dunnett’s test summary for intracellular and extracellular HRSA in *R. tetracirrata* exposed to Cd + Zn bimetallic mixtures compared with control. Values are expressed as mean ± SE.
