# Supplementary material for: Heavy Metals Affect the Antioxidant Defences in the Soil Ciliate Rigidohymena tetracirrata
Source: J Xenobiot. 2025 Oct 17;15(5):169. doi: 10.3390/jox15050169 (PMC12565027; doi:10.3390/jox15050169)
Supplement: Supplementary file 1 [file jox-15-00169-s001.zip › jox-3817272-supplementary.pdf]

Journal of Xenobiotics

MDPI

Supplementary Materials: Heavy Metals Affect the Antioxidant Defences in the Soil Ciliate *Rigidohymena tetracirrata*

Govindhasamay R. Varatharajan, Antonio Calisi, Santosh Kumar, Daizy Bharti, Arnab Ghosh, Shikha Singh, Amit C. Kharkwal, Martina Coletta, Francesco Dondero and Antonietta La Terza

Table S1. Dunnett's test summary for intracellular and extracellular TPC in *R. tetracirrata* exposed to single metals compared with control. Values are expressed as mean ± SE

Treatment	Intracellular TPC (Mean ± SE)	Mean Difference (vs. Control)	Significance	Cohen d	Extracellular TPC (Mean ± SE)	Mean Difference (vs. Control)	Significance	Cohen d
Control	33.73 ± 0.42	–	–	–	36.70 ± 0.39	–	–	–
Cd LC_{20}	65.82 ± 0.59	−32.08	***	44.39	49.25 ± 0.55	+12.55	***	18.78
Cd LC_{50}	61.50 ± 0.59	−27.77	***	38.41	43.45 ± 0.55	+6.75	***	10.10
Cu LC_{20}	44.84 ± 0.59	−11.11	***	15.378	60.18 ± 0.55	+23.47	***	35.13
Cu LC_{50}	50.12 ± 0.59	−16.39	***	22.67	53.17 ± 0.55	+16.46	***	24.64
Zn LC_{20}	56.68 ± 0.59	−22.94	***	31.75	48.15 ± 0.55	+11.44	***	17.13
Zn LC_{50}	59.02 ± 0.59	−25.28	***	34.98	58.28 ± 0.55	+21.57	***	32.28

Significance based on Dunnett's test: ***$p < 0.001$ for all treatments.

Table S2. Dunnett's test summary for intracellular and extracellular TPC in *R. tetracirrata* exposed to Cd + Zn bimetallic mixtures compared with control. Values are expressed as mean ± SE

Treatment (Cd + Zn)	Intracellular TPC (Mean ± SE)	Mean Difference (vs. Control)	Significance	Cohen d	Extracellular TPC (Mean ± SE)	Mean Difference (vs. Control)	Significance	Cohen d
Control	53.31±0.31	–	–	–	55.51±0.21	–	–	–
0.25 + 0.25	91.23 ± 0.43	+37.92	***	70.41	115.12 ± 0.61	+59.61	***	164.03
0.5 + 0.25	83.65 ± 0.37	+30.34	***	56.34	92.91 ± 0.09	+37.40	***	102.92
0.75 + 0.25	100.96 ± 0.62	+47.65	***	88.48	99.32 ± 0.39	+43.81	***	120.54
0.5 + 0.5	85.15 ± 1.02	+31.84	***	59.12	99.07 ± 0.17	+43.56	***	119.85
0.25 + 0.5	88.83 ± 0.80	+35.52	***	65.95	98.57 ± 0.15	+43.06	***	118.49
1 + 0.25	83.50 ± 0.29	+30.19	***	56.06	104.42 ± 0.07	+48.91	***	134.59
0.75 + 0.5	93.72 ± 0.27	+40.41	***	75.03	91.96 ± 0.61	+36.45	***	100.30
0.25 + 1	92.54 ± 0.62	+39.23	***	72.84	102.30 ± 0.13	+46.79	***	128.76

Significance based on Dunnett's test: ***$p < 0.05$ for all treatments.

Table S3. Dunnett's test summary for intracellular and extracellular DPPH in *R. tetracirrata* exposed to single metals compared with control. Values are expressed as mean ± SE

Treatment	Intracellular Mean ± SE	Mean Difference (vs. Control)	Significant	Cohen d	Extracellular Mean ± SE	Mean Difference (vs. Control)	Significant	Cohen d
Control	7.07 ± 0.41	–	–	–	3.07 ± 0.25	–	–	–
Cd LC_{20}	10.96 ± 0.59	–3.89	***	6.30	5.51 ± 0.25	+2.45	***	5.59
Cd LC_{50}	22.12 ± 0.31	–15.05	***	24.34	7.79 ± 0.25	+4.72	***	10.79
Cu LC_{20}	3.83 ± 0.39	+3.24	***	5.25	5.41 ± 0.25	+2.34	***	5.34
Cu LC_{50}	13.03 ± 0.46	–5.96	***	9.64	9.74 ± 0.25	+6.67	***	15.25
Zn LC_{20}	8.42 ± 0.83	–1.35	ns (0.077)	2.19	4.70 ± 0.25	+1.64	***	3.74
Zn LC_{50}	12.06 ± 0.29	–4.99	***	8.07	11.09 ± 0.25	+8.03	***	18.34

Significance based on Dunnett's test: ****p* < 0.001 for all treatments

Table S4. Dunnett's test summary for intracellular and extracellular DPPH in *R. tetracirrata* exposed to Cd + Zn bimetallic mixtures compared with control. Values are expressed as mean ± SE

Treatment (Cd + Zn)	Intracellular Mean ± SE	Mean Difference (vs. Control)	Significant	Cohen d	Extracellular Mean ± SE	Mean Difference (vs. Control)	Significant	Cohen d
Control	7.49±0.2	–	–	–	11.74 ± 0.75	–	–	–
0.25+0.25	45.68 ± 0.19	-38.19	***	115.01	40.87 ± 0.75	-29.13	***	22.53
0.5+0.25	43.21 ± 0.20	-35.72	***	107.58	45.66 ± 0.75	-33.92	***	26.24
0.75+0.25	42.58 ± 0.19	-35.09	***	105.66	37.21 ± 0.75	-25.46	***	19.70
0.5+0.5	27.63 ± 0.20	-20.14	***	60.65	49.59 ± 0.75	-37.84	***	29.27
0.25+0.5	29.40 ± 0.19	-21.91	***	65.98	47.83 ± 0.75	-36.09	***	27.91
1+0.25	42.01 ± 0.20	-34.52	***	103.97	52.08 ± 0.75	-40.34	***	31.20
0.75+0.5	35.11 ± 0.19	-27.62	***	83.16	55.73 ± 0.75	-43.98	***	34.02
0.25+1	31.77 ± 0.20	-24.28	***	73.12	50.56 ± 0.75	-38.81	***	30.02

Significance based on Dunnett's test: ****p* < 0.001 for all treatments.

Table S5. Dunnett's test summary for intracellular and extracellular HRSA in *R. tetracirrata* exposed to single metals compared with control. Values are expressed as mean ± SE

Treatment	Intracellular Mean ± SE	Mean Difference (vs. Control)	Significant	Cohen d	Extracellular Mean ± SE	Mean Difference (vs. Control)	Significant	Cohen d
Control	36.42±0.31	–	–	–	32.34±0.37	–47.29	–	–
Cd LC_{20}	31.65±0.31	–4.77	***	8.96	79.63±0.37	–48.60	***	74.05
Cd LC_{50}	61.10±0.31	+24.68	***	46.32	80.95±0.37	–44.14	***	76.11
Cu LC_{20}	72.67±0.31	–36.25	***	68.02	76.48±0.37	–37.56	***	69.11
Cu LC_{50}	73.64±0.31	–37.22	***	69.86	69.91±0.37	–24.51	***	58.82
Zn LC_{20}	71.52±0.31	–35.10	***	65.88	56.85±0.37	–6.04	***	38.38
Zn LC_{50}	68.25±0.31	–31.83	***	59.73	38.38±0.37	–47.29	***	9.45

Significance based on Dunnett's test: ****p* < 0.001 for all treatments.

Table S6. Dunnett's test summary for intracellular and extracellular HRSA in *R. tetracirrata* exposed to Cd + Zn bimetallic mixtures compared with control. Values are expressed as mean ± SE

Treatment (Cd + Zn)	Intracellular Mean ± SE	Mean Difference (vs. Control)	Significant	Cohen d	Extracellular Mean ± SE	Mean Difference (vs. Control)	Significant	Cohen d
Control	39.61 ± 0.14	–	–	–	33.39 ± 2.22	–	–	–
0.25+0.25	55.06 ± 0.04	-15.44	***	65.69	68.43 ± 2.22	-41.71	***	73.84
0.5+0.25	57.90 ± 0.07	-18.28	***	77.76	75.23 ± 2.22	-41.85	***	74.08
0.75+0.25	58.52 ± 0.11	-18.90	***	80.40	74.38 ± 2.22	-40.99	***	72.57
0.5+0.5	59.98 ± 0.08	-20.37	***	86.64	69.81 ± 2.22	-36.43	***	64.49
0.25+0.5	57.52 ± 0.11	-17.91	***	76.16	76.79 ± 2.22	-43.41	***	76.84
1+0.25	51.97 ± 0.14	-12.35	***	52.54	67.07 ± 2.22	-33.68	***	59.63
0.75+0.5	51.00 ± 0.16	-11.39	***	48.44	65.51 ± 2.22	-32.13	***	56.88
0.25+1	65.99 ± 0.05	-26.37	***	112.17	69.39 ± 2.22	-36.01	***	63.74

Significance based on Dunnett's test: ***$p < 0.001$ for all treatments.
